# Percutaneous transluminal angioplasty in the treatment of stenosis of arteriovenous fistulae for hemodialysis

**DOI:** 10.1186/1755-7682-1-16

**Published:** 2008-09-23

**Authors:** Daniel Gustavo Miquelin, Luis Fernando Reis, Adinaldo Adhemar Menezes da Silva, José Maria Pereira de Godoy

**Affiliations:** 1 Prof. of the Angiology and Vascular Surgery Service of the Medicine School in São Jose do Rio Preto (FAMERP), Brazil; 2 Livre Docente of the Cardiology and Cardiovascular Surgery Department of the Medical School in São José do Rio Preto (FAMERP), Professor of the Graduation and post graduation courses of FAMERP and CNPq researcher, Brazil

## Abstract

**Background:**

Thrombosis following stenosis of arteriovenous fistulae resulting in the loss of vascular access for hemodialysis is an important complication in patients with chronic renal failure. Percutaneous transluminal angioplasty is being used more frequently in the treatment of stenosis aiming at increasing the patency of arteriovenous fistulae.

**Objective:**

To evaluate the primary patency of arteriovenous fistulae following percutaneous transluminal angioplasty.

**Patients and method:**

Patients submitted to percutaneous transluminal angioplasty in the Angiology service of Hospital de Base in 2004 were analyzed over an average follow-up of 10 months (2 to 16 months).

**Results:**

A total of 22 angioplasties were performed in 20 fistulae of 19 patients. Of the 19 patients, one did not complete follow-up and one presented with a rupture of the fistulae. The following complications occurred in the remaining 18 fistulae, three deaths with two fistulae patent until death; one exeresis of prosthesis due to infection (53 days after the procedure); two thromboses (3 and 49 days after the procedure) and four restenosis (3 were submitted to a second angioplasty and one treated surgically). At the end of the follow-up, 11 fistulae (55%) were patent and with a flow rate in hemodialysis > 300 mL/min. Primary patency was 82.4% over three months; 81.2% over six months; 54.5% over 9 months and 50% over 1 year.

**Conclusion:**

Percutaneous transluminal angioplasty is an efficacious method for the correction of stenosis of arteriovenous fistulae for hemodialysis, prolonging the patency of the fistula and enabling new interventions.

## Introduction

The increase in the prevalence of patients with chronic renal failure on hemodialysis has made several measures to maintain the vascular access patent and efficacious necessary as, the morbimortality of these patients is directly related to the effectiveness of hemodialysis. This requires repeated punctures of large vessels capable of offering a rapid blood flow of adequate volume [1-3].

Thrombosis is the most frequent complication of arteriovenous fistulae that results in the loss of the access for hemodialysis. Most episodes of thrombosis coincide with the development of stenosis (in more than 85% of cases), generally located in the venous segment proximal to the arteriovenous anastomosis [4-6]. Stenosis in the arterial segment has been studied less but also compromises the patency of the vascular access, as well as resulting in ischemia of the limb and thus should be systematically investigated and identified in the dialytic population, generally composed of elderly patients with comorbidities, many of whom with generalized arterial disease[2,5]. A small percentage of the thrombosis is caused by hypotension, unintentional extrinsic pressure, trauma or infection [3].

Clinically, some findings may be suggestive of stenosis of the arteriovenous fistula such as the presence of segmental dilation along the route of the vein or its disappearance, persistent edema of the limb and the presence of a pulse without fremitis. Other indicators are evidenced by alterations during hemodialysis, such as inefficacious dialysis, low blood flow, increase in the venous pressure, difficulty to obtain another venous access, increase in the time necessary to reach hemostasis after hemodialysis, increase in the intra-access pressure and signs of recirculation. Thus, when diagnosed, intervention is justified to maintain the patency of the vascular access and consequently reduce the incidence of hemodialysis failure due to loss of the fistula [3,6,7].

Percutaneous transluminal angioplasty is a method that has proved to be efficacious in the treatment of stenosis to prolong the endurance of the fistulae [1,2].

The objective of the current study was to evaluate the primary patency of arteriovenous fistulae after percutaneous transluminal angioplasty.

## Patients and method

A total of 22 angioplasties of 20 fistulae in 19 patients (12 men and 7 women) with a mean age of 53.7 years (range 35 to 75 years) and on hemodialysis for chronic renal failure were analyzed. The angioplasties were performed in the angiology service of Hospital de Base in São José do Rio Preto between January 1 and December 31 2004.

The indications for angiographic studies of the arteriovenous fistula were based on clinical criteria or alterations detected during hemodialysis. The criteria utilized were an existent pulse but without fremitis, persistent edema of the limb with the fistula, the presence of constrictions and/or dilations on the route of the fistula, difficulty to insert the needles or hemostasis after their removal, blood flow at the access of less than 200 mL/min, increase in venous pressure and following thrombectomy surgery.

**Figure 1:**
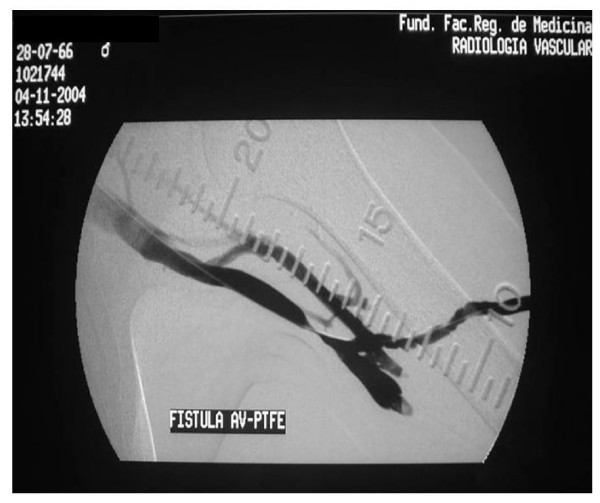
Angiographic study showing proximal stenosis at the anastomosis of the PTFE prosthesis and the axillary vein.

**Figure 2:**
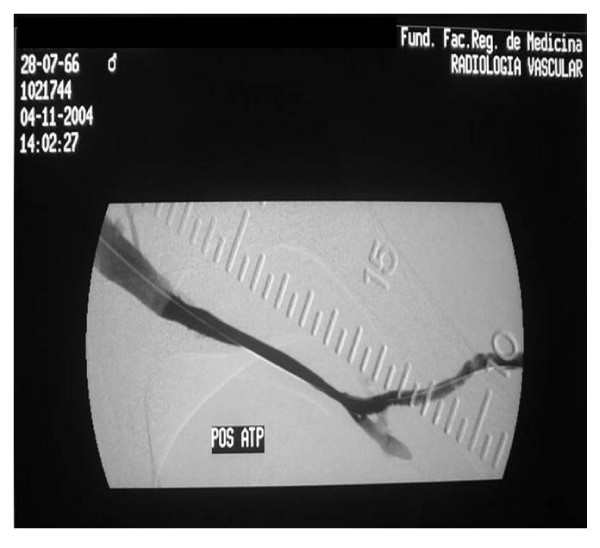
Angiographic control after balloon percutaneous transluminal angioplasty.

The angiographic technique employed in all cases was by venous puncture or puncturing the prosthesis using a 21 needle, the injection of contrast and obtaining digital angiographic images from the arteriovenous anastomosis up to the central veins. If significant stenosis was present, angioplasty was performed immediately (Figure 1 and 2).

The angioplasties were performed in all cases by proximal catheterization of the fistulae with the insertion of introductory sheaths (varying from 5 to 8 Fr) and regional anticoagulation using 2000 IU of heparin. The vessel was opened using a 0.35 mm × 260 cm hydrophilic guide wire (Terumo^®^) and dilated using a balloon catheter (Powerflex^®^, Ultra-thin Diamond^®^) with a diameter 1 mm greater than the normal size of the venous segment (varying from 4 to 8 mm). The balloon was maintained insufflated for 60 seconds and repeated when necessary using the pressure recommended by the manufacturer.

## Results

In the study period, 22 angioplasties were performed in 20 fistulae of 19 patients. Of these fistulae, 18 were with the cephalic or basilic veins and 2 with polytetrafluoroethylene (PTFE) prostheses.

Of the treated fistulae, one patient did not continue with the follow-up in the service after the angioplasty and one presented a rupture of the arteriovenous fistula during the procedure and was successfully submitted to surgical correction with implantation of a PTFE prosthesis.

The other 18 fistulae were evaluated after the procedure with a mean follow-up of 10 months (Range: 2 to 16 months). Of these, 17 fistulae presented a clinical follow-up of more than 3 months, 16 more than 6 months, 11 more than 9 months and 8 more than one year.

In this period, there were three deaths not related to the arteriovenous fistulae or the procedure, of which two were patients up to their deaths (120 and 289 days after the angioplasty) and one presented with thrombosis after 40 days and peritoneal dialysis was initiated.

One of the patients, 53 days after the angioplasty, presented with thrombosis of the fistula and was submitted to a thrombectomy with angioplasty, however he presented with an infection in the PTFE prosthesis which needed to be removed. In two other patients, thrombosis occurred with loss of the fistulae (with 3 and 49 post-procedure days).

During the follow-up, 4 cases of restenosis were diagnosed. Of these, 3 were submitted to a second angioplasty resulting in patent fistulae with flow rates for hemodialysis greater than 350 mL/min. One restenosis was surgically treated with the implantation of a PTFE prosthesis due to severe stenosis at multiple sites along the route of the vein (basilic).

At the end of the follow-up period, 11 fistulae were patent (11/20; 55%), with flow for hemodialysis greater than 300 mL/min.

The primary patency of the 18 fistulae evaluated after angioplasty was 82.35% over 3 months (14/17); 81.2% at 6 months (13/16); 54.5% at 9 months (6/11) and 50% over 1 year (4/8).

## Discussion

The current study shows that angioplasty is an efficacious method for the correction of stenosis of arteriovenous fistulae for hemodialysis and constitutes another therapeutic option. In the past, surgical treatment performing correction of the stenotic lesion using a bypass or a patch was the only option [3].

In recent years, several studies have demonstrated that angioplasty is efficacious with some advantages compared to the conventional surgical treatment such as a shorter time needed to perform the procedure and shorter hospitalization, less discomfort for the patient, and lower infection rates. Additionally, it enables dialysis immediately after the procedure without the necessity of using a central venous catheter [1-8].

Complications of angioplasty have been reported in about 2 to 16% of cases, with the most common being immediate venous rupture during the procedure, the formation of pseudoaneurysms, acute thrombosis and periprocedural bacteremia. Reaction to the contrast has not implicated a higher mortality rate of these patients [3,5]. In this study, 2 complications (9%) occurred: 1 venous rupture which was successfully treated by surgery and 1 case of acute thrombosis with loss of the fistula three days after angioplasty.

The literature shows that primary patency at 12 months after angioplasty is equivalent of surgical treatment [1]. The current study confirms these findings and suggests this therapeutic approach as another possible option for stenosis of arteriovenous fistulae.

## Conclusion

Angioplasty is efficacious in the correction of stenosis of fistulae for hemodialysis, increasing the survival rate of the fistula and allowing more interventions.

## Competing interests

The authors confirm the participation in study in all of the phases, that not of Conflicts of Interest and agree with the content of the manuscript. All authors certify that this manuscript was not published or by sending for publication in any form.

## Authors' contributions

DGM was responsible for surgical procedures and data collection and critically reviewed the manuscript. LFR was responsible for surgical procedures and data collection and critically reviewed the manuscript. AAMS was responsible for surgical procedures and data collection and critically reviewed the manuscript. JMPG conceived of the study, and participated in its design and coordination. All authors read and approved the final manuscript.
